# Enhanced Hydrophobicity, Thermal Stability, and X-Ray Shielding Efficiency of BaSO_4_/P(VDF-HFP) Nanocomposites for Advanced Lead-Free Radiation Protection

**DOI:** 10.3390/polym17060723

**Published:** 2025-03-10

**Authors:** Chaiporn Kaew-on, Jureeporn Yuennan, Nikruesong Tohluebaji, Phongpichit Channuie, Soraya Ruangdit, Ritiron Samran, Thanaphorn Tochomphoo, Ratchanewan Siri

**Affiliations:** 1 Faculty of Science and Technology, Nakhon Si Thammarat Rajabhat University, Nakhon Si Thammarat 80280, Thailand; chaiporn_kae@nstru.ac.th (C.K.-o.); jureeporn_yue@nstru.ac.th (J.Y.); 2 Faculty of Science and Technology, Princess of Naradhiwas University, Narathiwat 96000, Thailand; nikruesong.t@pnu.ac.th; 3 School of Science, Walailak University, Thasala, Nakhon Si Thammarat 80160, Thailand; 4 Demonstration School, Phetchaburi Rajabhat University, Phetchaburi 76000, Thailand; soraya.rua@mail.pbru.ac.th; 5 Nuclear Technology Research and Development Center, Thailand Institute of Nuclear Technology (Public Organization), Nakornnayok 26120, Thailand; ritiron@tint.or.th (R.S.); thanaphorn@tint.or.th (T.T.); 6 Division of Physical Science (Physics), Faculty of Science, Prince of Songkla University, Songkhla 90112, Thailand; ratchaneewan.s@psu.ac.th

**Keywords:** P(VDF-HFP) nanocomposites, barium sulfate particles, X-ray shielding

## Abstract

In this research, polymer composite sheets were developed by blending poly (vinylidene fluoride-co-hexafluoropropylene) or P(VDF-HFP) with varying concentrations of barium sulfate (BaSO_4_) for X-ray shielding applications. The photon counting technique was used to evaluate the composite shielding characteristics through the linear attenuation coefficient. Surface properties, including surface morphology, hydrophobicity, and surface energy, were analyzed using an atomic force microscope (AFM) and a water contact angle machine. Scanning electron microscopy (SEM) was employed to investigate the microstructural distribution and dispersion of BaSO_4_ particles within the polymer matrix, providing insights into the composite’s uniformity and structural integrity. Additionally, the bulk properties of the composite polymer sheets, such as crystal structures, tensile strength, and thermal stability, were examined. The results demonstrate that increasing the concentration of BaSO_4_ in BaSO_4_/P(VDF-HFP) composite sheets significantly improves their X-ray attenuation capabilities. Moreover, higher BaSO_4_ concentrations enhance the material’s hydrophobicity, flexibility, and thermal stability, highlighting the potential of these composites for advanced radiation shielding applications.

## 1. Introduction

X-rays, a form of ionizing radiation, pose a risk of causing damage to living tissue, particularly for patients and radiation workers [1]. It is necessary to protect against the danger posed by the radiation. The safety design aims to minimize damage caused by ionizing radiation by adhering to the ALARA principle, which emphasizes achieving the lowest reasonable level of exposure [2]. This principle involves considering factors such as time, distance, and shielding to ensure the safety of individuals and equipment. The traditional shielding material, lead, was favored due to its high density, atomic number, and low cost. However, it faced numerous drawbacks, including toxicity, weight, environmental disease, poor flexibility, and low chemical stability [3]. Lead-free materials emerge as alternative options, attracting numerous research groups to address the drawbacks associated with lead [4,5,6]. Polymer composite shielding has emerged as a promising lead-free material with significant potential for practical applications. Its advantages include lightweight design, excellent flexibility, and ease of processing [7]. Various polymers, such as polymethyl-methacrylate (PMMA), polyethylene terephthalate (PET), thermoplastic polyurethane (TPU), polyvinylpyrrolidone (PVP), and polyethylene glycol (PEG), have been employed as matrices for composite shielding materials [8,9,10,11,12]. Among these, poly(vinylidene fluoride) (PVDF) stands out as a fluorine-containing polymer known for its exceptional chemical and thermal resistance, mechanical strength, durability, and biocompatibility. PVDF’s high crystallinity significantly enhances its mechanical and thermal performance, making it a preferred choice for demanding applications [13]. However, this crystallinity also imposes limitations, such as reduced flexibility and processability, which restrict its use in adaptable applications. To address these challenges, poly(vinylidene fluoride-co-hexafluoropropylene) (P(VDF-HFP)) was developed as an advanced alternative. By incorporating hexafluoropropylene (HFP) into its polymer chain, P(VDF-HFP) reduces crystallinity compared to PVDF, resulting in improved flexibility and processability while retaining good mechanical strength [14]. These properties make P(VDF-HFP) a suitable candidate for applications that demand a balance between mechanical performance and adaptability, such as radiation shielding composites. Furthermore, P(VDF-HFP) exhibits greater hydrophobicity than PVDF due to the presence of trifluoromethyl (-CF_3_) groups in its molecular structure. These -CF_3_ groups not only increase the fluorine content but also lower the material’s surface energy, thereby enhancing its hydrophobic properties. This improved hydrophobicity provides greater resistance to moisture and minimizes the risk of microorganism colonization, a common issue in fluorine-containing polymers [15,16]. These combined features position P(VDF-HFP) as a versatile and advanced material for developing lead-free radiation shielding solutions that offer flexibility, moisture resistance, and high performance.

Barium sulfate (BaSO_4_) is a non-toxic, environmentally friendly compound with an effective atomic number of 104, making it highly effective for X-ray attenuation [17]. Commonly used in radiation shielding applications such as medical imaging and industrial settings, BaSO_4_ enhances polymer composites by improving impact resistance, chemical stability, heat resistance, dimensional stability, and mechanical strength. Its chemical stability and compatibility with polymers make it an excellent filler for composite development [18,19]. Although BaSO_4_’s incorporation into PVDF has been extensively studied [20,21], limited research has explored its integration into P(VDF-HFP) polymer matrices. To address this, lightweight, flexible, and water-resistant composite sheets made from BaSO_4_ and P(VDF-HFP) were developed in this study to provide efficient X-ray shielding. The linear attenuation coefficient was used to evaluate their shielding performance through photon counting techniques, while comprehensive analyses of surface morphology, hydrophobicity, crystal structures, mechanical properties, and thermal stability demonstrated their potential as advanced lead-free X-ray shielding materials.

## 2. Materials and Methods

### Materials and Film Preparation

In this study, the polymer matrix used was composed of 10 wt% P(VDF-HFP) (4.5 mol%) (Solef^®^ 11010/1001, Solvay, Bangkok, Thailand). N,N-dimethylformamide (DMF), a highly polar solvent (C_3_H_7_NO, D158550, Merck Ltd., Bangkok, Thailand), served as the solvent. Barium sulfate (BaSO_4_), a white powder with a molecular weight of 233.39 g/mol (KA264, Kemaus, New South Wales, Australia), was incorporated as the filler material.

**Figure 1:**
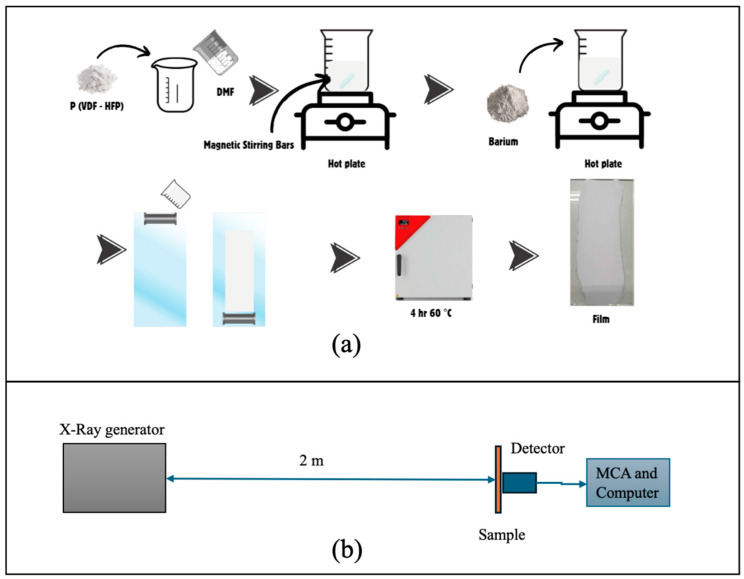
Schematic illustration of (**a**) the film preparation process and (**b**) the X-ray absorption experimental setup for BaSO_4_/P(VDF-HFP) composites.

To prepare the P(VDF-HFP) solution, 20 g of P(VDF-HFP) powder was dissolved in 100 mL of DMF at room temperature under constant stirring, resulting in a transparent solution (P(VDF-HFP)-DMF mixture) after complete dissolution. Various concentrations of BaSO_4_ (5, 10, 20, 30, and 40% *w*/*v*) were dispersed into the P(VDF-HFP) solution. For example, to prepare the 5% BaSO_4_/P(VDF-HFP) composite, 5 g of BaSO_4_ was added to 100 mL of the prepared P(VDF-HFP) solution, and the same method was applied for the other samples. The mixtures were magnetically stirred until homogenous. The resulting solutions were cast onto clean glass substrates and dried at 60 °C in a hot air oven (FD 115, Binder, Tuttlingen, Germany) for 4 h. Each sample was produced using approximately 5 mL of the prepared solution. Once dried, the pure P(VDF-HFP) and BaSO_4_/P(VDF-HFP) composite films were carefully peeled from the glass substrates. The thickness was measured using a dial thickness gauge (Peacock, PEA-G). The final film thickness was approximately 0.2 mm, making it suitable for subsequent characterization. The schematic illustration of the film fabrication process is presented in Figure 1a.

A. Sample characterization

1. Surface Morphology

The 2D surface morphology of all P(VDF-HFP) samples was analyzed using a scanning electron microscope (SEM; Quanta 400, FEI, Brno, Czech Republic) to visualize detailed surface features. Before imaging, the P(VDF-HFP) samples were carefully mounted onto SEM stubs using carbon tape to ensure stability and prevent charging under the electron beam. The surface microstructure was observed under standard high vacuum conditions, ensuring high-resolution imaging of the sample surfaces. A 20 kV accelerating voltage was applied, providing sufficient beam energy to interact with the sample surface, while a magnification of 5000× was used to capture fine details such as surface roughness, porosity, and any structural patterns. A porosity analysis of the surface morphology was conducted using ImageJ, a robust image analysis software developed by Wayne Rasband at the National Institutes of Health, USA, and available at https://imagej.net/ij/download.html (accessed on 22 February 2025). SEM images of the sample surface were processed in ImageJ to distinguish between porous and solid regions. The images were converted to grayscale and thresholded to generate a binary image, with pores represented in black color and solid areas in white color. By applying the “Analyze Particles” function, ImageJ (accessed on 22 February 2025) calculated the percentage of the surface area occupied by pores, providing a quantitative measure of porosity. This value reflects the distribution and size of pores across the sample surface, which is essential for understanding the material’s structural characteristics.

Atomic force microscopy (AFM) was used to examine the surface morphology and topography of P(VDF-HFP) films. The AFM images showed a smooth surface with nanoscale features. A 1 × 1 cm^2^ film was mounted on a stub and placed on the sample stage. The Nanosurf easyscan 2 Flex AFM system (Easyscan 2, Nanosurf AG, Liestal, Switzerland) was used in dynamic force mode, employing an ACL-A probe, suitable for non-contact or tapping mode. The analysis parameters were set at 50 μm for image size, 1 s per line, and 256 points per line. Surface roughness was analyzed using the Nanosurf software (Nanosurf easyScan2 Flex AFM system, Software version: 3.10.0.36, Firmware version: 3.8.1.0) with the roughness calculated through the root mean square roughness (*R_q_*), representing the standard deviation of surface height.

2. Hydrophobicity

Surface wettability is a crucial factor in ensuring the long-term performance of X-ray shielding materials, particularly in moisture-prone environments such as medical devices, protective garments, and industrial applications. Hydrophobic surfaces prevent water absorption, which helps maintain the mechanical integrity and shielding efficiency of the composite material over time. Moisture infiltration can degrade the polymer matrix, weaken mechanical strength, and reduce radiation attenuation efficiency. The contact angle and surface energy serve as key indicators of the wettability of the P(VDF-HFP) surface. In this study, the contact angle was measured using a Dataphysics contact angle goniometer (OCA-15EC, DataPhysics Instruments GmbH, Filderstadt, Germany) with SCA20_U software (Software version: 3.10.0.36, Firmware version: 3.8.1.0) in sessile drop mode. A 2 μL water droplet was used. A 1 × 3 cm^2^ film was placed on the sample holder, and a liquid droplet was deposited on the film’s surface. The droplet shape used for contact angle measurement is referred to as a sessile drop, which forms a spherical cap (a segment of a sphere) on the surface. The contact angle formed by the droplet was captured in an image for analysis. To minimize the experimental errors, the contact angles were measured at three random locations for each sample and then the average was reported. The water contact angle (WCA) on a rough surface can be calculated using the following equation [22]:

(1) $${cos\theta }_{CB}=f_{1}\left(cos\theta_{1}+1\right)-1,$$

where ${cos\theta }_{CB}$ is the Cassie-Baxter contact angle, $f_{1}$ is the fraction of the solid–liquid contact area, and $\theta_{1}$ is the contact angle of the droplet on the surface. Additionally, the surface energy (SE) of the membrane was analyzed using the Owens-Wendt model [23], based on the contact angles of water, formamide, and ethylene glycol.

3. Crystal Structures

The crystal structures of the samples were examined using Fourier Transform Infrared Spectroscopy (FTIR; Vertex70, Bruker Optics GmbH & Co. KG, Ettlingen, Germany) and X-ray Diffraction (XRD; Empyrean, PANalytical, Malvern Panalytical B.V., Almelo, The Netherlands). FTIR measurements were performed at room temperature in attenuated total reflectance (ATR) mode, with a spectral range from 4000 to 400 cm^−1^ and a resolution of 2 cm^−1^. The obtained spectra were analyzed following the methodology described in reference [24]. The XRD measurement was conducted using a Cu X-ray tube with an X-ray generator set to 40 kV and 0.3 mA, producing a wavelength of 0.154 nm (Cu Kα). The scan range was set from 5° to 90° (2θ) with a step size of 0.026° and a time per step of 70.125 s. The scanned XRD pattern is used to identify the crystalline regions in the sample through sharp diffraction peaks. The degree of the crystallinity (*Xc*) value was calculated from the peak areas in the obtained XRD patterns using the following equation [25]:

(2) $$X_{c}=\frac{\sum A_{cr}}{\sum A_{cr}+\sum A_{amr}}\times 100,$$

where Σ*A_cr_* and Σ*A_amr_* are the total integrated areas of the crystalline diffraction peaks and the amorphous halo, respectively. By integrating the peak areas, the relative amount of the crystalline material compared to the amorphous content is determined, providing the degree of crystallinity as a percentage.

4. Mechanical Property

To evaluate the mechanical properties of the P(VDF-HFP) copolymer, tensile tests were conducted using a Zwick Roell Germany testing machine (model z010) in accordance with the ISO 37-2017 standard [26]. Five samples were tested per composition, with each sample clamped at both ends and subjected to a 100 N load at a deformation rate of 5 mm/min at room temperature. The machine automatically measured stress, based on the applied force over the film’s cross-sectional area, as a function of elongation. Key mechanical properties such as tensile strength, elongation at break, and Young’s modulus were determined. Young’s modulus was calculated from the slope of the stress–strain curve in the elastic region, following Hooke’s law [27].

(3) $$Young’smodulus=\frac{Stress}{Strain}$$

5. Thermal Stability

The thermal stability of P(VDF-HFP) was investigated through thermogravimetric analysis (TGA) using the Perkin Elmer TGA 8000 instrument (Waltham, MA, USA). In this process, the sample was subjected to controlled heating, starting at 50 °C and gradually increasing to 700 °C, with a consistent heating rate of 10 °C per minute. The analysis was performed in an inert nitrogen atmosphere to prevent oxidation or combustion during heating. A constant nitrogen flow of 20 mL/min was maintained, ensuring that any evolved gases or decomposition products were carried away efficiently. This setup allowed for the precise monitoring of the sample’s weight loss, which provided insights into the thermal decomposition behavior and overall thermal stability of the P(VDF-HFP) samples over a broad temperature range.

6. Absorption performance

The equipment used to study X-ray absorption includes an X-ray generator (G.E. Titan E 320, GE Sensing&Inspection Technology, Ahrensberg, Germany), a semi-conductor detector CdTe (AMPTEX XR-100T, Amptek Inc., Bedford, MA, USA), and a multi-channel analyzer (MCA; AMPTEX PX5, Amptek Inc., Bedford, MA, USA). The composite film was set at 2 m from the X-ray source, as shown in Figure 1b. The distance between the X-ray source and the film was set to be relatively long to ensure an even distribution of radiation across the entire film. Furthermore, the use of radiation shielding materials for personnel working at a distance from the X-ray source is also taken into account. After generating an X-ray beam, the X-ray photon that transmits through the sample is detected by a detector and sends an electrical signal to be analyzed by MCA and the computer system. This process was repeated three times under each condition of each sample. The counts for each condition were averaged, and the total count was obtained by summing these averages. The properties of composite BaSO_4_/P(VDF-HFP) sheets on the attenuation performance were investigated from the linear attenuation coefficient (*μ*) that was calculated from the Beer–Lambert law as an equation [17]:

(4) $$I=I_{0}e^{-\mu x}.$$

By rewriting this equation, we obtain the following equation:

(5) $$\mu =\frac{1}{x}ln\frac{I_{0}}{I},$$

where $I_{0}$ and I are the incidents and transmitted intensity of X-ray, and x is the thickness of the sample. $I_{0}$ was evaluated from the average value of the X-ray intensity measurements when there is no shielding material. The source of the X-ray, together with a lead collimator, restricts the size and shape of the X-ray beam moving to the shielding film. From Equation (5), the linear attenuation coefficient (*μ*) was calculated from the slope of the linear equation, which contains $ln\frac{I_{0}}{I}$ and x, where $ln\frac{I_{0}}{I}$ is on the vertical axis and x on the horizontal axis.

To investigate the effect of the BaSO_4_ concentration in the BaSO_4_/P(VDF-HFP) composite sheet on the attenuation performance, the X-ray beam’s energy was generated at 60 keV and 0.1 mA. The transmitted intensities were collected from the intensity of X-rays that passed through the composite film with a thickness of 0.2 mm, 0.4 mm, 0.6 mm, 0.8 mm, 1.0 mm, and 1.2 mm. In contrast, the incident intensity was collected from the detector without any shielding. The thickness of 0.4 mm was made of 2 sheets of 0.2 mm films, and so on.

In addition, this research also investigates the effect of X-ray energy on the linear attenuation coefficient of composite shielding comprising BaSO_4_ at different concentrations in the P(VDF-HFP) polymer. The energy of the X-ray source was 60, 80, and 100 keV. The samples had a thickness of 0.2 mm for the BaSO_4_/P(VDF-HFP) composite at concentrations of 0, 5, 10, 20, 30, and 40%.

## 3. Results and Discussion

### 3.1. Surface Morphology

#### 3.1.1. SEM Analysis

**Figure 2:**
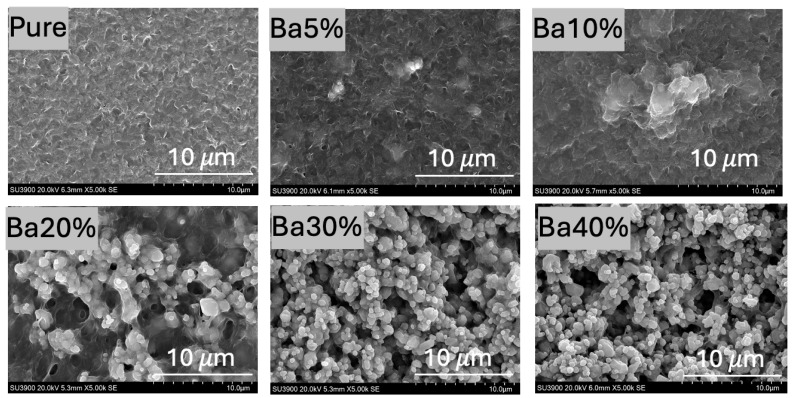
SEM micrographs of pure P(VDF-HFP) and BaSO_4_/P(VDF-HFP) composite films.

Figure 2 illustrates SEM micrographs showing the surface morphology of both pure P(VDF-HFP) and BaSO_4_/P(VDF-HFP) composite films. The micrograph of pure P(VDF-HFP) reveals a relatively smooth and uniform surface, with no visible filler particles or significant texture at the micro-scale. This smooth and dense morphology can be attributed to the solution casting method used during film fabrication, where the evaporation of the DMF solvent leaves behind a uniform polymer layer [28]. As BaSO_4_ was incorporated into the P(VDF-HFP) polymer matrix, the surface morphology becomes progressively rougher and more granular. At lower concentrations (5% BaSO_4_ to 10%BaSO_4_), the gradual addition of BaSO_4_ introduces small, scattered clusters of particles. The BaSO_4_ particles are generally more evenly dispersed and embedded within the polymer, allowing the matrix to maintain a relatively smooth surface, though some aggregation begins to appear at 10%BaSO_4_. This initial level of aggregation does not significantly disrupt the overall smoothness of the polymer. For 20%BaSO_4_ and higher, the surface becomes increasingly granular, with more pronounced clustering of BaSO_4_ particles. At higher concentrations (30%BaSO_4_ to 40%BaSO_4_), the particles are densely packed, resulting in rougher surfaces with agglomerated particles. The polymer matrix struggles to encapsulate and uniformly disperse the high volume of BaSO_4_, causing the particles to cluster together due to attractive forces, such as van der Waals interactions [14]. These aggregated clusters cannot be fully integrated into the polymer chains, leading to a granular and rough morphology. The agglomerates act as micro-sized protrusions, giving the surface its rough, grainy appearance.

#### 3.1.2. AFM Analysis

**Figure 3:**
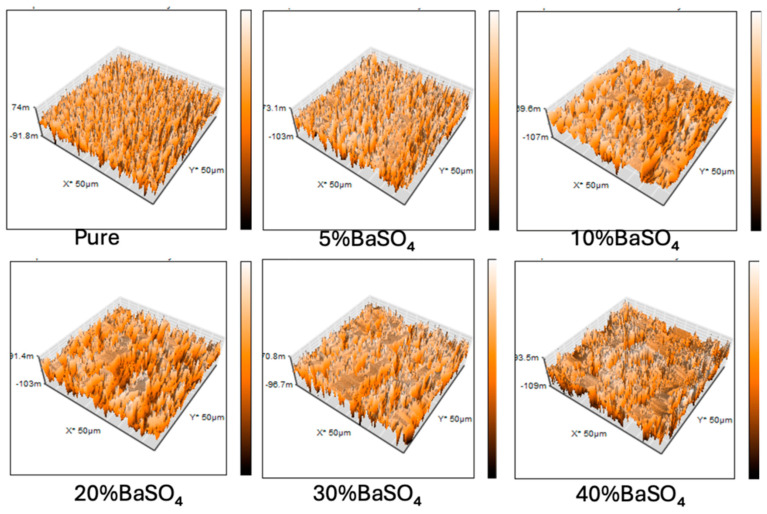
AFM images of pure P(VDF-HFP) and BaSO_4_/P(VDF-HFP) composite films with different BaSO_4_ concentrations from 5–40%.

AFM analysis was conducted to further support the surface morphology observations. The AFM images reveal that the BaSO_4_/P(VDF-HFP) composite films exhibit significant surface roughness on a large scale. Figure 3 presents the AFM images of pure P(VDF-HFP) and BaSO_4_/P(VDF-HFP) composite films, and the corresponding surface roughness values (Rq) are listed in Table 1. The AFM image of pure P(VDF-HFP) shows a relatively smooth surface with minimal height variation. The surface roughness is quite low (Rq: 66.57 nm), which is consistent with the SEM micrographs and the previously reported morphology of pure P(VDF-HFP) films. This smoothness suggests that in the absence of BaSO_4_ fillers, the polymer chains form a dense and uniform film during the solution casting process. It also indicates that the polymer matrix can crystallize uniformly when there is no filler particles present to disrupt the structure. For BaSO_4_/P(VDF-HFP) composites, surface roughness increases with BaSO_4_ content. The results clearly show a progressive increase in surface roughness as the BaSO_4_ content rises from 5% to 40%. This is due to the growing number of BaSO_4_ particles in the polymer matrix, which raises the likelihood of particle aggregation, leading to an uneven topography.

At lower concentrations of BaSO_4_, such as 5% to 10%, the AFM images reveal a slight increase in surface roughness compared to the pure polymer. Small bumps or granular formations appear, indicating the presence of dispersed BaSO_4_ particles. However, the surface remains relatively smooth, suggesting that the BaSO_4_ particles are well dispersed and have not significantly disrupted the uniformity of the polymer matrix. At 20%BaSO_4_, the *Rq* value (180.66 nm) increases significantly compared to the 10% BaSO_4_/P(VDF-HFP) sample, indicating that larger clusters have formed, introducing greater height variations and resulting in a rougher surface, as also shown in the SEM results. At higher concentrations (30% to 40%), surface roughness is dominated by BaSO_4_ particle agglomerates, as the polymer matrix can no longer maintain an even dispersion of the filler. This results in a highly irregular and rough surface texture. The polymer’s capacity to encapsulate and uniformly disperse BaSO_4_ is exceeded at these concentrations, leading to large-scale agglomeration and the formation of surface protrusions. This change in morphology can affect the microstructural and mechanical properties of the composite films, which is important to consider for x-ray absorption applications.

### 3.2. Hydrophobicity

For X-ray shielding materials, surface wettability can impact their performance, particularly in environments where moisture exposure is a concern (e.g., medical devices and protective clothing). Hydrophobic surfaces may resist moisture better, protecting the integrity of the shielding material over time. The water contact angle (WCA) measures how water interacts with a surface. A WCA below 90° indicates hydrophilicity, between 90° and 150° indicates hydrophobicity, and above 150° indicates superhydrophobicity, reflecting how well a surface repels or attracts water [29]. WCA is inversely related to surface energy (SE), which influences a material’s behavior, including adhesion, durability, and interaction with other materials [30].

**Figure 4:**
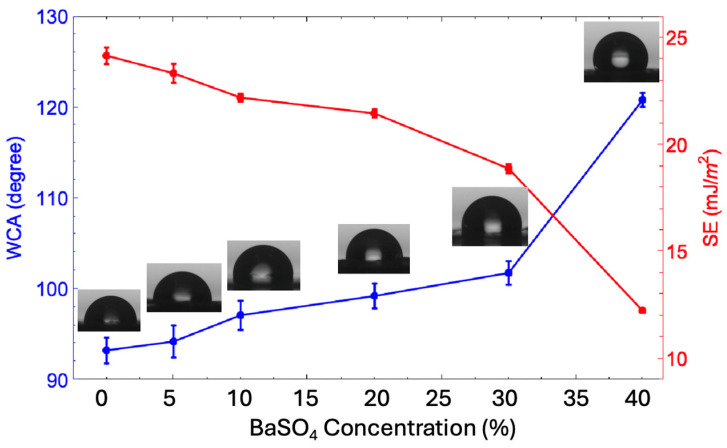
Water contact angle (WCA) and surface energy (SE) of pure P(VDF-HFP) and BaSO_4_/P(VDF-HFP) composite films with different BaSO_4_ concentrations from 5–40%.

Figure 4 presents the WCA and SE results for pure P(VDF-HFP) and BaSO_4_/P(VDF-HFP) composite films. For pure P(VDF-HFP), the WCA is 93.28° ± 1.42°, and the SE is 24.18 ± 0.39 mJ/m^2^, indicating a slightly hydrophobic surface. The relatively high SE contributes to moderate wettability. This confirms the hydrophobic nature of P(VDF-HFP), attributed to the presence of CF_3_ and fluorine atoms, which make fluorine-containing polymers naturally hydrophobic. Additionally, SEM in the previous section showed a smooth polymer surface with minimal roughness, as expected in a uniform polymer matrix. AFM would likely confirm minimal nanoscale roughness, consistent with the relatively low WCA. As BaSO_4_ was incorporated (from 5% to 20%), the WCA gradually increased from 94.26° ± 1.42° to 99.27° ± 1.37°, suggesting a slow shift toward greater hydrophobicity. Simultaneously, the SE decreased steadily from 23.36 ± 0.44 to 21.48 ± 0.19 mJ/m^2^, implying that BaSO_4_ particles were lowering the surface tension. SEM revealed dispersed BaSO_4_ particles within the polymer matrix, likely contributing to slight surface roughness. AFM also indicated a subtle increase in nanoscale roughness, correlating with the gradual rise in WCA. For the composite with the highest BaSO_4_ content (40%), the WCA reached 120.85° ± 0.47°, and SE dropped to 12.25 ± 0.06 mJ/m^2^, indicating significant hydrophobicity approaching superhydrophobic behavior. Although BaSO_4_ itself is hydrophilic [31], which would normally decrease WCA and increase SE, the observed behavior can be attributed to the substantial aggregation of BaSO_4_ particles, as revealed by SEM and AFM images. These particles likely formed rough micro- and nanoscale structures, increasing surface roughness, which ultimately enhanced WCA and reduced SE. This outcome can be explained by Young’s and Wenzel’s equations, both of which relate surface roughness to wettability [32]. Young’s equation describes the equilibrium contact angle (θ) for a liquid droplet on an ideal, smooth surface by taking into account interfacial free energies (surface tensions) between solid, liquid, and gas phases. However, this model assumes a homogeneous (for pure P(VDF-HFP)), flat surface, making it less applicable to rough surfaces like those observed in BaSO_4_/P(VDF-HFP) composites. The Wenzel equation, an extension of Young’s model, considers surface roughness by introducing a roughness factor, which amplifies the natural wetting behavior of a surface. According to the Wenzel model, for hydrophobic surfaces, increased roughness leads to higher contact angles as the liquid follows the contours of the rough surface [33]. Thus, the high BaSO_4_ content created an uneven, rough surface that amplified the material’s hydrophobicity, as predicted by the Wenzel model, even though BaSO_4_ itself is hydrophilic. This suggests that surface morphology, rather than the inherent chemical nature of BaSO_4_, dominated the wettability properties of the composite, driving it towards greater hydrophobicity.

### 3.3. Crystal Structure

#### 3.3.1. FTIR Analysis

**Figure 5:**
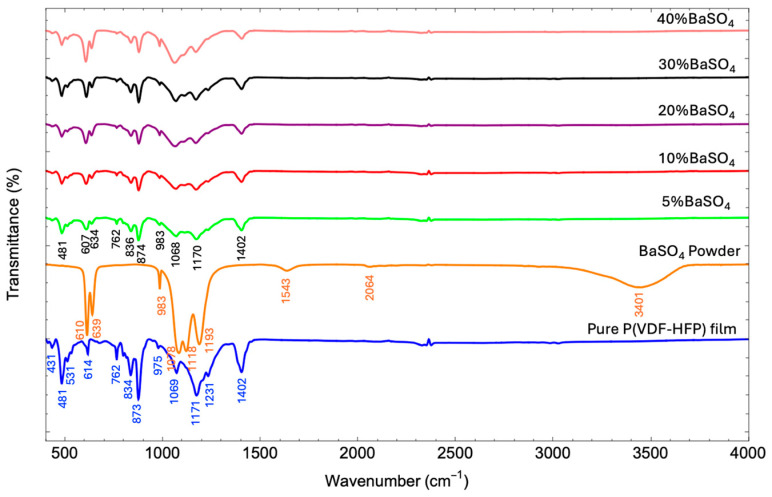
FTIR patterns of pure P(VDF-HFP) and BaSO_4_P(VDF-HFP) composite films with different BaSO_4_ concentrations from 5–40%.

FTIR spectroscopy is a crucial tool for analyzing the crystal structure of polymers, enabling the optimization of mechanical and thermal properties in X-ray shielding materials. By assessing phase composition and crystallinity, FTIR analysis supports the development of durable, efficient materials that meet strict safety and performance standards for radiation protection. Figure 5 shows FTIR peaks associated with the crystal structures of both P(VDF-HFP) and BaSO_4_ in the composites. For pure P(VDF-HFP), vibrational peaks are observed at 431 cm^−1^, 481 cm^−1^, and 531 cm^−1^ (CF_2_ bending); 614 cm^−1^ (CF_2_ bending and skeletal bending); 762 cm^−1^ (in-plane bending/rocking); 834 cm^−1^ (CH_2_ rocking and CF_2_ asymmetric stretching); 873 cm^−1^ (CH_2_ rocking); 975 cm^−1^ (CH out-of-plane deformation); 1069 cm^−1^ (CF_3_ out-of-plane deformation); 1171 cm^−1^ (CF_2_ antisymmetric stretching); 1231 cm^−1^ (CF out-of-plane deformation); and 1402 cm^−1^ (CH_2_ scissoring). These peaks confirm the semi-crystalline nature of P(VDF-HFP), with both amorphous and crystalline regions contributing to its structure [34,35,36]. For the BaSO_4_ powder, the sulfate group exhibits four fundamental vibrational modes: one nondegenerate mode (ν_1_), one doubly degenerate mode (ν_2_), and two triply degenerate modes (ν_3_ and ν_4_). The FTIR spectrum of BaSO_4_ typically displays several significant bands, with intense bands associated with asymmetric stretching and bending (ν_3_ and ν_4_) and weaker bands representing symmetric stretching and bending (ν_1_ and ν_2_). In the FTIR spectrum, bands in the range of 1078–1193 cm^−1^ and a shoulder at 983 cm^−1^ correspond to the symmetric stretching of the SO_4_^2−^ group. The SO_4_^2−^ group’s stretching vibration is identified at 1543 cm^−1^ (ν_3_), while peaks at 610 and 639 cm^−1^ correspond to out-of-plane bending. The absorption peak at 3401 cm^−1^, attributed to the H_2_O stretching vibrations at vacant Ba sites, is evident in pure BaSO_4_ but absent in the composites, suggesting the elimination of hydration-related vibrations during composite formation. Peaks around 2064 cm^−1^ represent overtones and combinations of stretching and bending of the sulfur–oxygen bond powder [37,38].

In the FTIR spectra of BaSO_4_/P(VDF-HFP) composites with varying BaSO_4_ concentrations, characteristic peaks from both the P(VDF-HFP) polymer matrix and BaSO_4_ are retained, indicating the successful integration of BaSO_4_ within the polymer structure. Key BaSO_4_ peaks, including SO_4_^2−^ bending vibrations at 610 and 639 cm^−1^ and symmetric stretching vibrations within the range of 1078–1193 cm^−1^, remain stable across all concentrations, underscoring the sulfate group’s structural integrity in the composite. As BaSO_4_ concentration increases, these specific peaks grow in intensity, reflecting the increasing BaSO_4_ content. Meanwhile, prominent P(VDF-HFP) polymer peaks, such as CF_2_ bending at 531 and 614 cm^−1^, CF_2_ in-plane bending or rocking at 762 cm^−1^, and CH_2_ rocking at 834 cm^−1^, are preserved across all composite spectra. This suggests that BaSO_4_ does not significantly disrupt the semi-crystalline structure of P(VDF-HFP), allowing the composite to retain the structural and mechanical properties of the polymer [21]. Additionally, peak shifts observed in the FTIR spectra of the composites may indicate changes in the chemical environment around specific functional groups due to interactions between BaSO_4_ and the P(VDF-HFP) matrix. When BaSO_4_ is incorporated into P(VDF-HFP), the particles interact with the polymer chains, potentially altering local electronic distributions and bond strengths within the polymer. Such interactions could cause slight shifts in the CF_2_ bending or CH_2_ rocking peaks of P(VDF-HFP), reflecting molecular-level interactions that affect the polymer’s structural arrangement. These shifts provide valuable insights into the compatibility and interaction between the phases within the composite material [20]. The absence of the 3401 cm^−1^ peak, associated with H_2_O stretching vibrations in the pure BaSO_4_ powder, suggests that hydration in BaSO_4_ is eliminated during composite formation, likely due to thermal processing or polymer–particle interactions that reduce the presence of free water molecules. This elimination of water content is advantageous as it enhances the composite’s durability and thermal stability, making it suitable for applications in environments where moisture could affect material performance. Overall, the composite retains the structural integrity of P(VDF-HFP) alongside the stability of BaSO_4_, highlighting its balanced integration, mechanical flexibility, and suitability for radiation shielding applications.

#### 3.3.2. XRD Analysis

**Figure 6:**
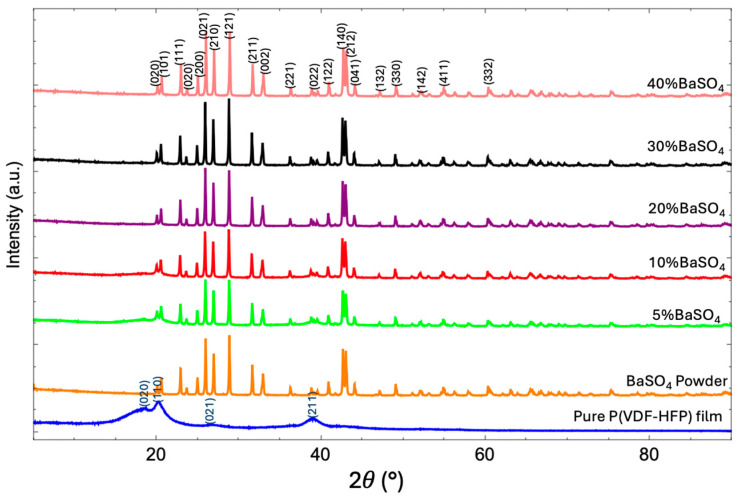
XRD patterns of pure P(VDF-HFP) and BaSO_4_P(VDF-HFP) composite films with different BaSO_4_ concentrations from 5–40%.

The crystalline data for pure P(VDF-HFP) and BaSO_4_/P(VDF-HFP) composites provide key insights into how BaSO_4_ loading affects the overall crystalline structure of the composite material. Figure 6 shows the XRD intensity data at various 2θ angles for pure P(VDF-HFP), the BaSO_4_ powder, and BaSO_4_-P(VDF-HFP) composite films with different BaSO_4_ concentrations. Table 1 lists the crystallinity levels evaluated from XRD results using Equation (2). P(VDF-HFP) consists of alternating segments of vinylidene fluoride (VDF) and hexafluoropropylene (HFP), with a structural formula represented as follows: [—CH_2_—CF_2_—]_n_—[—CF_2_—CF(CF_3_)—]_m_, where n and m represent the relative ratios of the VDF and HFP units in the copolymer. The P(VDF-HFP) structure combines crystalline (from VDF) and amorphous (from HFP) regions, resulting in a semi-crystalline copolymer that can exhibit multiple crystalline phases, primarily the α, β, and γ phases, depending on the polymer chain configuration [39]. The XRD pattern of the pure P(VDF-HFP) film reveals broad, low-intensity peaks, indicating its semi-crystalline structure. These peaks, associated with the crystalline α-phase of P(VDF-HFP), are relatively weak and broad, reflecting a moderate crystallinity level of 60.1% [40]. This semi-crystalline structure combines rigid crystalline domains and flexible amorphous regions. However, with light elements like carbon, hydrogen, and fluorine, pure P(VDF-HFP) has limited X-ray shielding capability due to its low atomic number composition. To investigate the BaSO_4_ powder, its XRD pattern reveals sharp, high-intensity peaks at specific 2θ values, reflecting its crystalline structure. According to Sifontes’s research [37], BaSO_4_ powder typically exhibits prominent, sharp peaks in its diffraction pattern, a characteristic of a crystalline material. This pattern confirms BaSO_4_’s orthorhombic crystal structure and highlights its high degree of crystallinity. The recorded XRD data display peaks at defined 2θ positions, particularly between 10° and 80°, which correspond to characteristic diffraction angles of BaSO_4_. The peak intensity underscores the material’s purity and structural integrity, distinguishing it from less crystalline or amorphous forms. Additionally, the sharpness and intensity of these peaks indicate the stable sulfate ion arrangement within the lattice, which is essential for applications requiring materials with high thermal stability and low reactivity.

The XRD patterns of BaSO_4_/P(VDF-HFP) composites reveal that increasing BaSO_4_ content impacts the crystalline structure of the P(VDF-HFP) matrix. When BaSO_4_ is incorporated, changes in both XRD patterns and crystallinity demonstrate its influence on the composite’s structure. Additional peaks, a characteristic of BaSO_4_’s crystalline structure, appear in the XRD patterns, confirming its successful integration and dispersion within the matrix. The broad α-phase peak of P(VDF-HFP) around 2θ ≈ 20° is absent, indicating that slight changes in intensity suggest structural modifications within the polymer matrix. As BaSO_4_ concentration rises, the XRD peaks sharpen and intensify, particularly around BaSO_4_’s characteristic angles, indicating an increase in overall crystallinity. This effect is likely due to BaSO_4_ acting as a nucleating agent, promoting the formation of crystalline domains within the P(VDF-HFP) matrix. This nucleation enhances polymer chain organization, leading to a more crystalline structure. The resulting higher crystallinity improves material rigidity and stability, which is beneficial for applications requiring a more robust composite material. Furthermore, the inclusion of BaSO_4_, with its high atomic number, increases the composite’s density and enhances its potential for X-ray attenuation. Higher BaSO_4_ concentrations improve X-ray shielding, as evidenced by the persistent BaSO_4_ peaks in the XRD patterns, which confirm its presence and distribution within the matrix. The broadening and reduction in P(VDF-HFP) peaks contribute to effective X-ray absorption and shielding. This increased density and enhanced X-ray shielding make these composites suitable for lightweight radiation protection applications.

### 3.4. Mechanical Property

**Figure 7:**
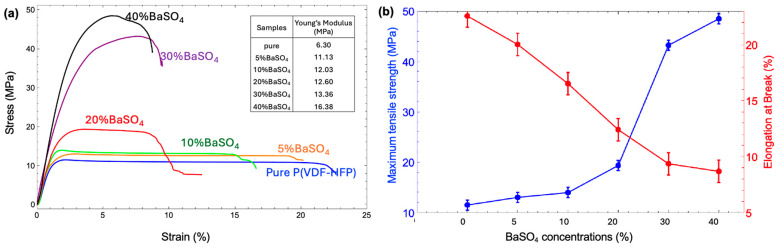
(**a**) Stress–strain curves and Young’s modulus (inserted table). (**b**) Values of tensile properties (tensile strength and elongation at the break) of pure P(VDF-HFP) and BaSO_4_/P(VDF-HFP) composite films with different BaSO_4_ concentrations from 5–40%.

The mechanical properties of polymer composite films are crucial for practical applications, especially in contexts such as X-ray shielding, where both strength and flexibility are required. Adding a BaSO_4_ filler to P(VDF-HFP) significantly affects these properties. To achieve optimal mechanical performance, it is essential to control the BaSO_4_ content and ensure uniform particle dispersion within the composite. Standard tensile tests reveal the impact of BaSO_4_ on tensile properties, including Young’s modulus, tensile strength, and elongation at breaks, as illustrated in Figure 7. For pure P(VDF-HFP), the stress–strain curve displays an initial elastic phase, followed by a peak stress that transitions into plastic deformation, with minimal resistance to further stretching. This behavior is a characteristic of a ductile polymer. The tensile properties show a Young’s modulus of 6.3 MPa, indicating low stiffness and high flexibility. With a tensile strength of 11.59 MPa and an elongation at a break of 22.61%, pure P(VDF-HFP) is highly flexible but lacks mechanical strength due to the absence of reinforcement. Upon introducing BaSO_4_, the stress–strain curves of the 5–20% BaSO_4_/P(VDF-HFP) composites retain a similar shape to that of pure P(VDF-HFP), but with enhanced stiffness and tensile strength. As BaSO_4_ content increases, both Young’s modulus and tensile strength rise, reaching 12.6 MPa and 19.45 MPa, respectively, at 20% BaSO_4_. The elongation at the break, however, decreases from 20.07% to 12.45%, indicating reduced ductility as the material stiffens. These composites offer a balance between strength and flexibility, making them suitable for X-ray shielding applications where moderate stiffness and improved tensile strength are advantageous. For the 30–40% BaSO_4_/P(VDF-HFP) composites, the stress–strain curves indicate substantial increases in tensile strength and rigidity, showcasing the strong reinforcing effect of BaSO_4_. With Young’s modulus values of 13.4 MPa and 16.4 MPa, as well as tensile strengths reaching 43.33 MPa and 48.59 MPa for 30% and 40% BaSO_4_, respectively, these composites demonstrate high load-bearing capacity. However, the elongation at the break falls to 9.41% and 8.73%, signifying increased brittleness and decreased flexibility. These properties make the 30–40% BaSO_4_ composites ideal for applications requiring robust strength and rigidity, although they may be less suitable for environments needing flexibility. As noted by Xiaolei Chen et al. [18] in studies on BaSO_4_-filled polymer composites, the Young’s modulus and tensile strength of these materials increase progressively, while elongation decreases with higher BaSO_4_ content. When inorganic particles are embedded within a polymer matrix, stress can transfer from the matrix to the particles, thereby enhancing yield stress. The tensile yield stress in particle-filled polymers is primarily influenced by filler content and interfacial interactions, which depend on factors such as interfacial adhesion, particle size, and dispersion within the matrix. BaSO_4_ particles, in particular, benefit from their hydrophobic surface, which allows for better dispersion in the polymer matrix. Consequently, as BaSO_4_ content rises, the interface transfers more stress from the matrix to the inorganic particles, leading to increased tensile yield stress in the composite.

The mechanical properties of the BaSO_4_/P(VDF-HFP) composites were influenced by the loading of BaSO_4_, with higher filler content generally leading to reduced flexibility and mechanical strength. Based on our results, composites with 20–30% BaSO_4_ provided an optimal balance between mechanical strength and X-ray shielding performance. This loading is suitable for applications where both mechanical durability and radiation protection are required, such as wearable protective equipment or flexible shielding panels. In contrast, composites with 40% BaSO_4_ exhibited superior shielding performance but compromised mechanical flexibility, making them more appropriate for stationary shielding applications where mechanical flexibility is less critical. This balance between mechanical properties and shielding effectiveness highlights the importance of selecting the appropriate BaSO_4_ loading based on the specific requirements of the intended application.

### 3.5. Thermal Stability

**Figure 8:**
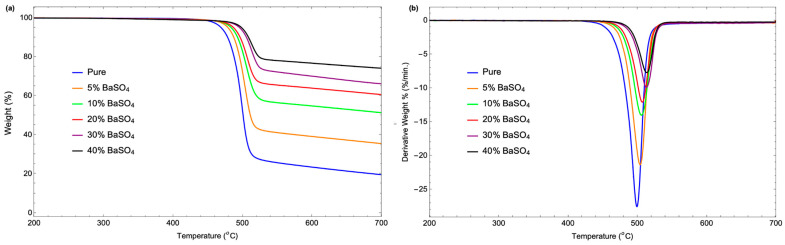
(**a**) TGA and (**b**) DTG thermograms of pure P(VDF-HFP) and BaSO_4_/P(VDF-HFP) composite films with different BaSO_4_ concentrations from 5–40%.

The thermal stability evaluation of P(VDF-HFP) and BaSO_4_/P(VDF-HFP) composites for X-ray shielding involves key measurements such as the decomposition temperature, weight loss rate, and residual mass. A high decomposition temperature and a low weight loss rate indicate heat resistance, while a stable residual mass reflects BaSO_4_’s durability as a filler. This analysis helps optimize BaSO_4_ content to enhance thermal resilience and ensure reliable shielding performance. The thermogravimetric (TGA) and derivative thermogravimetric (DTG) curves for pure P(VDF-HFP) and BaSO_4_/P(VDF-HFP) composite films, as shown in Figure 8 and summarized in Table 2, reveal differences in the decomposition temperature, weight loss rate, and residual mass between the pure polymer and the composites. From Figure 8a, pure P(VDF-HFP) begins significant weight loss around 469 °C, indicating relatively low thermal stability. In contrast, BaSO_4_/P(VDF-HFP) composites display higher decomposition temperatures, starting at approximately 479 °C for the 5% BaSO_4_ composite and reaching around 498 °C for the 40% BaSO_4_ composite. Additionally, the weight loss rate decreases with increased BaSO_4_ content, indicating slower degradation in composites. For instance, the 30% BaSO_4_ composite shows a more gradual weight loss, demonstrating BaSO_4_’s heat-resistant effect, which stabilizes the polymer matrix and delays degradation. Furthermore, the residual mass after thermal decomposition is minimal for pure P(VDF-HFP), as it fully degrades, leaving little residue. However, BaSO_4_/P(VDF-HFP) composites retain progressively higher residual mass with increased BaSO_4_ content, with the 40% BaSO_4_ composite showing the highest residual mass. This residual mass is mainly due to the thermally stable BaSO_4_, which remains intact and provides a durable framework even after the polymer matrix has decomposed, enhancing the composite’s suitability for high-temperature applications.

In Figure 8b, the DTG data provide additional information on the thermal decomposition behavior of pure P(VDF-HFP) and BaSO_4_/P(VDF-HFP) composites, specifically the peak decomposition temperature, maximum weight loss rate, and final residual mass for each sample. The peak decomposition temperature, where each sample experiences the maximum weight loss rate, is around 499 °C for pure P(VDF-HFP), indicating lower thermal stability compared to the composites. With increasing BaSO_4_ content, the peak decomposition temperature gradually rises, reaching 504 °C for the 5% BaSO_4_ composite, 507 °C for the 20% BaSO_4_ composite, and 513 °C for the 30% BaSO_4_ composite. This increase in peak temperature with higher BaSO_4_ content suggests that BaSO_4_ stabilizes the polymer matrix, delaying thermal decomposition. The maximum weight loss rate, representing the steepest part of the decomposition curve, is highest for pure P(VDF-HFP) at 27.55%/min, suggesting rapid degradation under thermal stress. As BaSO_4_ content increases, the weight loss rate decreases significantly, with the 5% BaSO_4_ composite at 21.36%/min and the 40% BaSO_4_ composite at 7.73%/min, indicating improved resistance to sudden thermal breakdown. The residual mass represents the material left after heating to the final temperature, indicating the stability of the inorganic BaSO_4_ component. Pure P(VDF-HFP) has negligible residual mass, as it fully degrades without leaving a stable residue. In contrast, BaSO_4_/P(VDF-HFP) composites retain progressively higher residual mass with increased BaSO_4_ content. For instance, the 5% BaSO_4_ composite has a small residual mass, while the 30% BaSO_4_ composite retains a much larger amount. This residual mass is primarily composed of thermally stable BaSO_4_, which does not decompose and remains intact, providing structural stability even after polymer degradation. These results indicate that increasing BaSO_4_ content in P(VDF-HFP) composites raises the peak decomposition temperature, reduces the maximum weight loss rate, and increases the final residual mass, demonstrating BaSO_4_’s role in enhancing the thermal stability and durability of the composite for high-temperature X-ray shielding applications requiring prolonged structural integrity.

### 3.6. Absorption Performance

**Figure 9:**
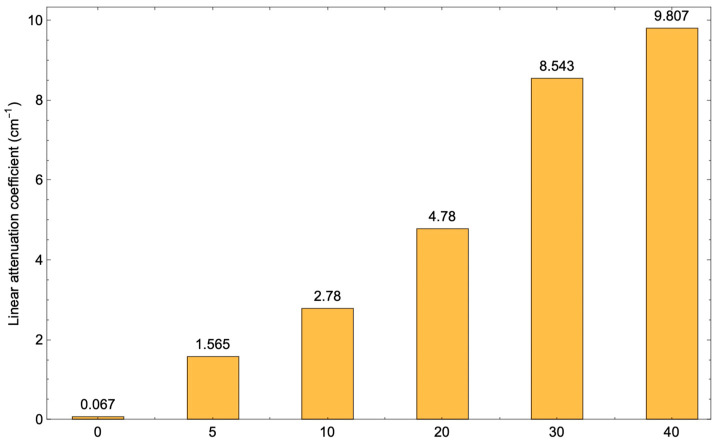
Relation between BaSO_4_ concentration in the PVDF-HFP composite film and the linear attenuation coefficient (μ).

The linear attenuation coefficient is a parameter that is used to describe the fraction of attenuated incident X-ray photons per unit thickness of the material. In general, the primary interactions within shielding materials encompass the photoelectric effect, Compton scattering, and pair production [41]. However, pair production occurs when the energy of the incident photon exceeds 1.022 MeV. Since this study employed low-energy photons (60–100 keV), the potential interaction mechanisms should be photoelectric and Compton scattering. In the case of the photoelectric effect, all the energy of the incident photon is absorbed by an atomic electron, unlike Compton scattering, where the energy transferred to an electron depends on the scattering angle [42]. The linear attenuation coefficient (μ), a crucial factor in evaluating the absorption capacity of shielding materials, is influenced by several key variables. These include the atomic number of the shielding material, its physical density, and the energy of the incident X-ray photon. BaSO_4_ has a high effective atomic number. The increase in BaSO_4_ concentration was observed to enhance the linear attenuation coefficient, as illustrated in Figure 9. Consequently, the corresponding value of X-ray photon intensity after traversing samples with varying BaSO_4_ concentrations is presented in Table 3. Each value of μ was calculated from the slope of the linear equation $ln\frac{I_{0}}{I}$ and the thickness of the shielding (x). The results show that the increase of BaSO_4_ causes the linear attenuation coefficient to increase. This phenomenon occurs because there is an increased likelihood that X-ray photons will collide with electrons within the atoms of barium sulfate, resulting in the photoelectric effect.

Additionally, from the evaluation of the mass attenuation coefficient (μ/ρ) of the shielding film composed of BaSO_4_ at concentrations of 0, 5, 10, 20, 30, and 40, the values obtained were 0.08, 0.43, 7.29, 12.77, 11.38, and 10.26 cm^2^/g, respectively. The results showed that increasing the concentration of BaSO_4_ increases the mass attenuation coefficient, reaching its maximum value at a BaSO_4_ concentration of 20%. Further increases in concentration result in a decrease in the mass attenuation coefficient. This may be due to the uneven distribution and increased porosity within the film, which are confirmed by analyzing the microstructure of the samples using SEM to check for agglomeration or porosity and verify the uniformity of BaSO_4_ dispersion. The study on BaSO_4_/P(VDF-HFP) composites was compared with BaSO_4_/PVC and BaSO_4_/PS composites from S. Jaiyen et al. [43] to evaluate X-ray shielding performance. Both studies showed that attenuation efficiency increases with BaSO_4_ content up to an optimal level, beyond which it declines due to structural factors. BaSO_4_/P(VDF-HFP) achieved a peak mass attenuation coefficient of 12.77 cm^2^/g at 20% BaSO_4_, significantly higher than BaSO_4_/PVC (4.599 cm^2^/g at 20%) and BaSO_4_/PS (2.362 cm^2^/g at 20%). Additionally, while BaSO_4_/PVC and BaSO_4_/PS composites peaked at 60% BaSO_4_, BaSO_4_/P(VDF-HFP) required only 20% BaSO_4_ for maximum shielding, making it more efficient at lower filler content. The decline in attenuation at higher BaSO_4_ concentrations in BaSO_4_/P(VDF-HFP) was attributed to agglomeration and porosity, whereas in BaSO_4_/PVC and BaSO_4_/PS, it was due to density saturation and dispersion issues. From a practical perspective, BaSO_4_/P(VDF-HFP) composites demonstrate superior shielding efficiency, flexibility, and hydrophobicity, making them well-suited for applications such as wearable shielding, flexible radiation barriers, and protective coatings. In contrast, BaSO_4_/PVC and BaSO_4_/PS composites are more appropriate for rigid shielding structures. The findings suggest that a 20% BaSO_4_ loading optimally balances shielding effectiveness and material durability, minimizing the risk of degradation under prolonged radiation exposure.

The radiation attenuation performance of BaSO_4_/P(VDF-HFP) composites was analyzed by evaluating the percentage attenuation, which was determined from the difference between the intensity of the incident and transmitted X-ray photons, as shown in Table 4. The results indicate that both BaSO_4_ concentration and shielding thickness significantly influence X-ray attenuation efficiency. The highest attenuation percentage was achieved with a 40% BaSO_4_ concentration and a 1.2 mm thickness under an X-ray source of 60 keV and 0.1 mA. These findings suggest that optimizing BaSO_4_ loading and film thickness enhances shielding effectiveness, while excessive filler content may introduce microstructural variations that influence performance.

The investigation of the percentage attenuation of the shielding at different concentrations of BaSO_4_ in BaSO_4_/P(VDF-HFP) shielding sheets by the high energy of X-ray photons is shown in Table 5. These results show that the ability of the shielding decreases when radiating with high energy. It is possible that the main part of X-ray photons was scattered and moved through the shielding. This phenomenon leads to a decrease in the linear attenuation coefficients of the shielding.

## 4. Conclusions

Polymer composite shielding offers an alternative approach to mitigate the risk of X-ray radiation exposure. In this method, a selected polymer acts as a matrix, while a high atomic number particle serves as a filler to absorb the X-ray photons. BaSO_4_/P(VDF-HFP) composite shielding sheets were produced with varying concentrations of BaSO_4_. Post irradiation with X-ray photons, these sheets were analyzed to assess their photon absorption capacity, surface characteristics, and bulk properties. The results indicated that increasing the concentration of BaSO_4_ in BaSO_4_/P(VDF-HFP) enhances the linear attenuation coefficient of the shielding sheet when exposed to 60 keV and a 0.1 mA X-ray generator. However, the linear attenuation coefficient decreases with higher X-ray generator power at 80 and 100 keV. Additionally, higher BaSO_4_ concentrations led to rougher and more hydrophobic surfaces on the shielding sheets. The WCA measurements demonstrated that surface roughness, influenced by BaSO_4_ content, significantly impacts the hydrophobicity of the composite films. Higher BaSO_4_ concentrations led to increased surface roughness, resulting in improved hydrophobicity, which is beneficial for long-term application in moisture-exposed environments. This interplay between surface roughness and wettability highlights the practical importance of our findings in developing durable and effective lead-free X-ray shielding materials. Furthermore, tensile and thermal tests showed an improvement in the shielding sheet’s properties.

## Figures and Tables

**Table 1:** Porosity, root mean square roughness, crystallinity values of pure P(VDF-HFP) and BaSO_4_/P(VDF-HFP) composite films.

Sample	Porosity (%)	Root Means Square Roughness (*R_q_*) (nm)	Crystallinity (*X_c_*) (%)
Pure	0.30	66.57	60.10
5% BaSO_4_	1.60	138.93	71.24
10% BaSO_4_	4.67	139.96	73.81
20% BaSO_4_	15.05	180.66	79.00
30% BaSO_4_	20.40	237.45	80.44
40% BaSO_4_	25.21	590.32	83.53

**Table 2:** Decomposition temperature, weight loss rate, and residual mass evaluated from TGA and DTG thermograms of pure P(VDF-HFP) and BaSO_4_/P(VDF-HFP) composite films.

Sample	TGA Analysis Results			DTG Analysis Results		
	Decomposition Temperature (°C)	Weight Loss Rate (%/°C)	Residual Mass (%)	Peak Decomposition Temperature (°C)	Maximum Weight Loss Rate (%/min)	Residual Mass (%)
pure	468.87	0.55	19.58	499.09	27.55	−0.06
5% BaSO_4_	478.78	0.46	35.50	503.72	21.36	−0.07
10% BaSO_4_	483.38	0.34	51.34	505.7	14.03	−0.28
20% BaSO_4_	486.69	0.27	60.60	506.86	12.10	−0.16
30% BaSO_4_	495.29	0.24	66.15	512.65	9.93	−0.19
40% BaSO_4_	498.26	0.18	74.11	513.15	7.73	−0.23

**Table 3:** The intensity of X-ray photons after passing through a shielding film with varying concentrations of BaSO_4_, along with the initial intensity of 210,954 cps and the X-ray energy of 60 keV.

BaSO_4_ (%)	Thickness (cm)					
	0.02	0.04	0.06	0.08	0.10	0.12
0	211,070	210,862	209,526	209,749	210,277	209,406
5	207,830	200,578	194,822	188,871	181,691	178,249
10	200,517	192,060	180,053	170,326	160,278	153,130
20	187,772	170,768	155,874	144,114	128,009	116,125
30	169,853	133,643	111,235	95,902	81,458	71,207
40	163,051	126,983	105,969	87,571	72,480	60,077

**Table 4:** Attenuation of X-ray photons due to the component of BaSO_4_ in BaSO_4_/P(VDF-HFP) composites with different BaSO_4_ concentrations from 5–40%.

Thickness (cm)	Attenuation (%)				
	5%BaSO_4_	10%BaSO_4_	20%BaSO_4_	30%BaSO_4_	40%BaSO_4_
0.02	1.48	4.95	10.99	19.48	22.71
0.04	4.92	8.96	19.05	36.65	39.81
0.06	7.65	14.65	26.11	47.27	49.77
0.08	10.47	19.26	31.68	54.54	58.49
0.10	13.87	24.02	39.32	61.39	65.64
0.12	15.50	27.41	44.95	66.25	71.52

**Table 5:** Attenuation of X-ray photons due to the component of BaSO_4_ in BaSO_4_/P(VDF-HFP) composites at different X-ray photon energy.

X-Ray Energy (keV)	Attenuation (%) of the 0.2 mm Thickness				
	5%BaSO_4_	10%BaSO_4_	20%BaSO_4_	30%BaSO_4_	40%BaSO_4_
60	1.48	4.95	10.99	19.48	22.71
80	2.28	2.12	7.33	10.96	13.95
100	0.04	1.20	3.74	6.29	7.89

## Data Availability

The original contributions presented in this study are included in the article. Further inquiries can be directed to the corresponding author.
